# Heat-inactivated Factor B inhibits alternative pathway fluid-phase activation and convertase formation on endothelial cell-secreted ultra-large von Willebrand factor strings

**DOI:** 10.1038/s41598-023-33007-3

**Published:** 2023-04-08

**Authors:** Nancy A. Turner, Joel L. Moake

**Affiliations:** Department of Bioengineering, Rice University, Houston, TX USA

**Keywords:** Innate immunity, Mechanisms of disease

## Abstract

Defective regulation of the alternative complement pathway (AP) causes excessive activation and promotes the inflammation and renal injury observed in atypical hemolytic-uremic syndrome (aHUS). The usefulness of heat-inactivated Factor B (HFB) in reducing AP activation was evaluated in: fluid-phase reactions, using purified complement proteins and Factor H (FH)-depleted serum; and in surface-activated reactions using human endothelial cells (ECs). C3a and Ba levels, measured by quantitative Western blots, determined the extent of fluid-phase activation. In reactions using C3, FB, and Factor D proteins, HFB addition (2.5-fold FB levels), reduced C3a levels by 60% and Ba levels by 45%. In reactions using FH-depleted serum (supplemented with FH at 12.5% normal levels), Ba levels were reduced by 40% with HFB added at 3.5-fold FB levels. The effectiveness of HFB in limiting AP convertase formation on activated surfaces was evaluated using stimulated ECs. Fluorescent microscopy was used to quantify endogenously released C3, FB, and C5 attached to EC-secreted ultra-large VWF strings. HFB addition reduced attachment of C3b by 2.7-fold, FB by 1.5-fold and C5 by fourfold. Our data indicate that HFB may be of therapeutic value in preventing AP-mediated generation of C3a and C5a, and the associated inflammation caused by an overactive AP.

## Introduction

The alternative complement pathway (AP) is initiated in the fluid phase when Factor B (FB) binds to hydrolyzed C3 (C3-H_2_O). Following the cleavage of FB by Factor D (FD), C3-H_2_O-Bb complexes are formed^1,2^. The Bb in these complexes proceeds to cleave additional C3 to C3b (releasing C3a) to form transient C3bFB complexes. FB is rapidly cleaved by FD (releasing Ba) to produce the C3 convertase, C3bBb. The AP regulatory protein, Factor H (FH) prevents any unwanted C3 convertase formation by displacing either FB or Bb that is bound to C3b or to C3-H_2_O. In the absence of FH, C3 convertases react rapidly with C3 and FB, resulting in unrestricted generation of C3a and Ba^3,4^.

The fluid-phase generated C3b indiscriminately binds to surfaces through an internal thioester that is exposed in C3 after its cleavage by Bb. The extent of C3b surface deposition and further activation, depends on whether the surface is an activator of the AP. C3 convertase formation on host, non-activating surfaces, are either prevented with the displacement of FB by FH, or bound C3b are inactivated by Factor I (FI) cleavage in conjunction with FH. On foreign surfaces of invading microorganisms, further deposition of C3b near previously formed C3bBb complexes, promotes C5 binding and formation of C5 convertases (C3bBbC3b). Convertases on surfaces with higher densities of C3b preferably bind C5 and induce the formation of terminal membrane attack complexes (C5b-9)^5^. In addition to fluid regulators, FH and FI, host cells have membrane-bound complement regulatory proteins to prevent and inactivate convertases formed on membrane surfaces. Human endothelial cell (ECs) produce both the complement components and the associated fluid-phase and surface regulatory proteins necessary to control complement over-activation^6–12^.

Defective regulation of the AP causes excessive activation and promotes the inflammation, microvascular thrombosis, and renal endothelial injury observed in atypical hemolytic-uremic syndrome (aHUS)^13,14^. A major cause of aHUS is deficiency of functional FH resulting from gene mutations or acquired function-blocking antibodies^15,16^. Normally, FH prevents fluid-phase AP activation and the formation of activation complexes on host cell surfaces^3^. Impairment of additional regulatory proteins, including FI, the five FH-related proteins, CD46 (membrane cofactor protein), and CD141 (thrombomodulin) have also been associated with development of aHUS^17–20^. Additionally, gain-of-function mutations in C3 and CFB, producing protein complexes more resistant to regulation, have been reported^13,21–23^.

To promote hemostasis and thrombosis, ECs secrete multimeric ultra-large von Willebrand factor (ULVWF) in hyper-adhesive long string-like structures that instigate platelet adhesion^24,25^. ECs also secrete ULVWF strings in response to stimulation by various agents, including histamine, lipopolysaccharide, and inflammatory cytokines^26^. In situations of excessive EC stimulation and inadequate cleavage by ADAMTS-13 (the VWF-specific protease), ULVWF strings persist on EC surfaces and participate in the accumulation of complement proteins and activation of the AP^12,27^. The AP response is exacerbated in conditions of deficiencies in functional complement regulatory proteins. Many studies have also shown connections between reduced levels of ADAMTS-13 activity in conjunction with AP regulatory dysfunction in patients with aHUS^28–33^.

In this study, we evaluated the effect of heat-inactivated FB (HFB) as a new type of agent to modulate AP activation. We investigated the use of HFB to reduce AP activation in early fluid-phase reactions; and additionally, to restrict the assembly of C3/C5 convertases on EC-secreted ULVWF multimeric strings.

## Results

### AP activation in fluid-phase reactions

#### Validation of linear range detection in Western blot analyses

Western blot analysis was used to measure C3a and Ba generated in fluid-phase reactions as indicators of AP activation. Prior to experimental analyses, several protein concentrations of C3, C3a, FB, and Ba were tested using Western blots to determine the range of linear signal detection. Western blot membranes with lanes containing 7.5 to 240 ng/lane of reduced C3 or C3a-desArg (C3a) were analyzed, using fluorescent intensity measurement for both total protein and specific C3/C3a antibody detection. Reduced dilutions of C3 verified that the entire range C3 protein levels were within the linear range of detection using either the total protein stain or anti-C3a antibody detection (although the C3a antibody detects only the C3 alpha chain, but not the C3 beta chain). C3a lane amounts of 60 ng and below were linearly proportional to signal intensities in both types of measurements (Supplemental Fig. S1).

In parallel verification experiments, the antibody used to detect FB and Ba proteins was tested for linear detection. FB and Ba proteins, ranging from 7.5 to 240 ng per lane, were analyzed by specific FB antibody and for total protein. Total protein intensities were linear across the entire range of FB and Ba amounts. FB antibody detection resulted in linear correlations between protein amounts and measured intensities with maximums of 30 ng of FB and 60 ng of Ba per lane (Supplemental Fig. S1).

#### The extent of AP activation is FB-dependent

AP cleavage products Bb, Ba and C3a were measured in reactions composed of C3, FB and FD in EGTA/MgCl_2_ buffer for 15 min at 37 °C. Concentrations of C3 (60 ng/µl) and FD (0.07 ng/µl) were constant, using normal serum ratios, whereas the normal concentration of FB (10 ng/µl) was either decreased by twofold (to 5 ng/µl), or increased by twofold (to 20 ng/µl) (Supplemental Table S1). Decreased FB concentrations, without changes in C3 or FD, resulted in decreased generation of each AP activation product, Bb, Ba and C3a (Supplemental Fig. S2). Signal intensities measured from Western blots for C3, C3a, Bb and Ba, were linearly correlated with changes in FB concentration. Ba and C3a mean intensities were correlated significantly with decreases in FB band intensities (FB and Ba, P = 0.017; FB and C3a, P = 0.0065, using Pearson’s correlation coefficient). C3 levels increased in reactions with lower amounts of FB because less C3a was generated and more C3 remained intact (Supplemental Fig. S2).

#### Heat-inactivated FB (HFB) inhibits activation of the AP

The extent of AP activation was quantified on Western blots that included a range of C3a and Ba protein levels (to generate standard curves) in order to interpolate values (Supplementary Fig. S3). Levels of C3a and Ba were measured quantitatively on Western blots using this method in the subsequent fluid-phase AP reactions. FB was purified from human plasma and inactivated by controlled heating to produce heat-inactivated FB (HFB)^34^. The heating process impaired the enzymatic function of HFB, although the binding capacity of HFB to C3b was preserved. HFB inhibits AP activation by competing effectively with FB for binding to C3b and forming complexes (C3b-HFB) incapable of supporting further activation.

**Figure 1:**
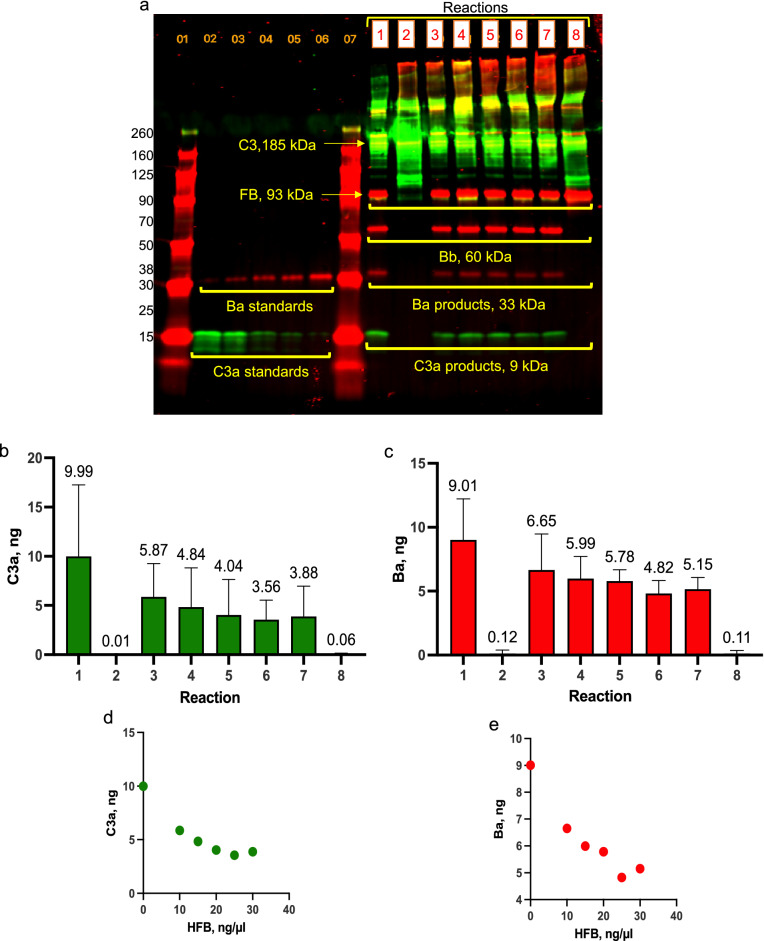
AP activation was reduced by addition of heat-inactivated FB. Levels of C3a and Ba, generated in AP reactions containing purified proteins with increasing amounts of HFB, were measured by quantitative Western blot analysis. Reactions, labeled with boxed numbers 1–8, consisted of: (1) 10.4 ng/µl FB + C3 + FD (without HFB); (2) 10.4 ng/µl HFB + C3 + FD (without FB); (3) 10.4 ng/µl HFB + 10.4 ng/µl FB + C3 + FD; (4) 15.6 ng/µl HFB + 10.4 ng/µl FB + C3 + FD; (5) 20.8 ng/µl HFB + 10.4 ng/µl FB + C3 + FD; (6) 26 ng/µl HFB + 10.4 ng/µl FB + C3 + FD; (7) 31.2 ng/µl HFB + 10.4 ng/µl FB + C3 + FD; and (8) 10.4 ng/µl HFB + 10.4 ng/µl FB + C3, without FD. C3 concentration is 60 ng/µl and FD is 0.07 ng/µl in each reaction, except for reaction 8. (**a**) Representative blot of merged detection of C3/C3a using rabbit anti-C3a + donkey anti-rabbit IRDye-800 (green); and FB/Bb/Ba using goat anti-FB + donkey anti-goat IRDye-680 (red). C3a and Ba standards are in lanes 2–6, and protein ladders are in lanes 1 and 7. Graphs of reaction (**b**) C3a levels; and (**c**) Ba levels are means plus SD from 3–5 experiments. Values were not statistically different by 1-way ANOVA with Tukey’s multiple comparisons test. (**d**,**e**) Correlation plots of HFB concentrations and generated C3a, r^2^ = 0.9224 (**d**); and Ba, r^2^ = 0.9509 (**e**). Pearson’s coefficients of determination (r^2^) were calculated for HFB concentrations from 0 to 26 ng/µl.

In the 15 min, 37 °C reactions, C3, FB and FD levels remained constant (at normal serum ratios) and concentrations of HFB ranged from 10.4 to 31.2 ng/µl (1-, 1.5-, 2-, 2.5-, and 3-fold higher than reaction levels of FB at 10.4 ng/µl) (Table 1). In reactions with HFB substituted for FB, bands for C3a and Ba were mostly undetectable in Western blots (Fig. 1a, reaction 2) and there was minimal production of C3a (Fig. 1b) and Ba (Fig. 1c). The absence of these activation products indicated that HFB was incapable of activating C3 (to C3b) and that FD was unable to cleave and activate HFB to Bb. In the presence of FB, generation of both AP activation products, C3a and Ba, were reduced with increasing concentrations of HFB. Mean C3a levels generated without HFB addition were reduced by 60% with the addition of 26 and 31.2 ng/µl HFB (Fig. 1b), and Ba was decreased by 45% with these HFB concentrations (Fig. 1c). Only negligible amounts of C3a and Ba were generated in control reactions with the omission of FD (reaction 8) (Fig. 1b,c**)**. Correlation plots show that the generation of AP activation products C3a and Ba decreased linearly with increasing concentrations of HFB until a plateau was reached with 26 ng/µl HFB (Fig. 1d,e). Although the measured changes of C3a and Ba with HFB addition in these reactions are not statistically significant (using 1-way ANOVA), the difference between mean values suggests that addition of HFB reduced AP activation.

**Table 1:** Concentrations of AP proteins and HFB in reactions using purified complement components.

Reaction description	Reaction concentration, ng/µl				HFB to FB ratio
	FB	HFB	C3	FD	
1. 10.4 ng/µl FB, C3, FD	10.4	none	60	0.07	
2. 10.4 ng/µl HFB, C3, FD	none	10.4	60	0.07	
3. 10.4 ng/µl HFB, FB, C3, FD	10.4	10.4	60	0.07	1-fold
4. 15.6 ng/µl HFB, FB, C3, FD	10.4	15.6	60	0.07	1.5-fold higher
5. 20.8 ng/µl HFB, FB, C3, FD	10.4	20.8	60	0.07	2-fold higher
6. 26 ng/µl HFB, FB, C3, FD	10.4	26	60	0.07	2.5-fold higher
7. 31.2 ng/µl HFB, FB, C3, FD	10.4	31.2	60	0.07	3-fold higher
8. No FD, HFB, FB, C3	10.4	10.4	60	none	1-fold

The description summarizes the proteins included in the reaction and the changes in HFB concentration. Reaction concentrations for each protein are in the center columns. The HFB to FB ratio describes changes in HFB concentrations to FB concentration. The reactions were in EGTA/MgCl_2_ buffer and activation occurred over 15 min at 37 °C. The results are shown in Fig. 1.

### AP activation in Factor H-depleted (FH-D) serum

#### AP activation measured in FH-D serum with FH addition

The absence of FH in the FH-D serum was first confirmed using blot analysis. The blots were analyzed for FH detection, and also for the abundant serum protein, vitronectin (240–540 mg/ml). Detection of vitronectin allowed the serum amounts to be visualized in lanes that appeared blank using FH detection antibodies. FH signals were below the limits of detection in each FH-D serum dilution tested, whereas vitronectin detection was positive (Supplemental Fig. S4). (Vitronectin, also known as S protein, functions as a fluid-phase regulatory protein in the complement terminal pathway)^35^.

**Figure 2:**
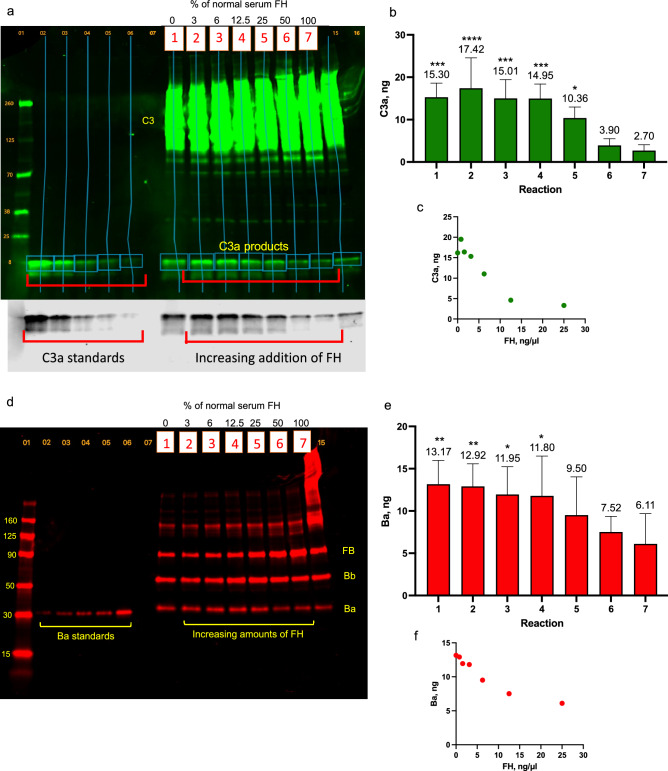
Increasing concentrations of FH reduced AP activation in FH-D serum. AP activation was induced in FH-D serum by the addition of Mg^+2^ ions in the presence of increasing concentrations of FH. C3a and Ba levels were measured by quantitative Western blot analysis. The representative blot was detected for: (**a**) C3a, using rabbit anti-C3a + donkey anti-rabbit IRDye-800 (green), (the lower section in grayscale shows a clearer view of the C3a standards and C3a activation products); and in (**d**) Ba, using goat anti-FB + donkey anti-goat IRDye-680 (red). The protein ladder is in lane 1, C3a and Ba standards are in lanes 2–6, and the reaction samples, labeled with boxed numbers 1–7, are in lanes 8–14. The sample in lane 15 was not included in the analysis. Graphs show the quantification of C3a (**b**) and Ba (**e**); and correlation plots of reaction FH concentrations with (**c**) C3a levels; and (**f**) Ba levels. Data are means + SD from 3–4 experiments. Statistical significance in (**c**) and (**f**) is shown for C3a and Ba levels in reactions 1–5 compared to levels in reactions 6 and 7; *P < 0.05, **P < 0.005, ***P < 0.0005, ****P < 0.0001. Statistics were analyzed by 2-way ANOVA with Tukey’s multiple comparisons test. P values for C3a: 1 vs. 6, P = 0.0001; 1 vs. 7, P < 0.0001; 2 vs. 5, P = 0.02; 2 vs. 6, P < 0.0001; 2 vs. 7, P < 0.0001; 3 vs. 6, P = 0.0002; 3 vs. 7, p < 0.0001; 4 vs. 6, P = 0.0002; 4 vs. 7, P < 0.0001; 5 vs. 6, P = 0.041; 5 vs. 7, P < 0.0001; and for Ba: 1 vs. 6, P = 0.0022; 1 vs. 7, P = 0.0002; 2 vs. 6, P = 0.0033; 2 vs. 7, P = 0.0003; 3 vs. 6, P = 0.0167; 3 vs. 7, P = 0.0016; 4 vs. 6, P = 0.0217; 4 vs. 7, P = 0.002.

In FH-D serum reactions, the serum was diluted in 0.1 mM EDTA to produce 60 ng/µl C3 and 10.4 ng/µl FB. AP activation products, C3a and Ba, were measured in reactions containing FH-D serum, 15 min after the addition of increasing concentrations of FH (3 to 100% normal serum levels) in MgCl_2_/EGTA buffer (Table 2). Levels of both C3a (Fig. 2a,b) and Ba (Fig. 2d,e) decreased with increasing FH. FH concentrations above 12.5 ng/µl (50% and 100% normal serum levels in reactions 6 and 7), resulted in significantly reduced generation of both activation products compared to FH-D serum without FH addition (in reaction 1) (Fig. 2b, C3a: 1 vs. 6, P = 0.0001; 1 vs. 7, P < 0.0001; and Fig. 2e, Ba: 1 vs. 6, P = 0.0022; 1 vs. 7, P = 0.0002). There was a linear, negative correlation between the extent of AP activation and the FH concentration, upward to 12.5 ng/µl of FH, and a leveling at the maximum FH level of 25 ng/µl (Fig. 2c, C3a; Fig. 2f, Ba).

**Table 2:** FH additions and concentrations in FH-D serum reactions.

Reaction description	FH levels in reactions	
	FH, ng/µl	% Normal serum levels
1. FH-D serum alone	0	
2. FH-D serum + 0.78 ng/µl FH	0.78	3
3. FH-D serum + 1.56 ng/µl FH	1.56	6
4. FH-D serum + 3.13 ng/µl FH	3.13	12.5
5. FH-D serum + 6.25 ng/µl FH	6.25	25
6. FH-D serum + 12.5 ng/µl FH	12.5	50
7. FH-D serum + 25 ng/µl FH	25	100

AP activation was measured in FH-depleted serum reactions with the addition of increasing amounts of FH. Table 3 shows the final FH concentrations in the FH-D serum reactions, along with comparable FH levels in normal serum. C3a and Ba levels were measured after 15 min at 37 °C. The results are in Fig. 2.

#### AP activation in FH-D serum with HFB addition

**Figure 3:**
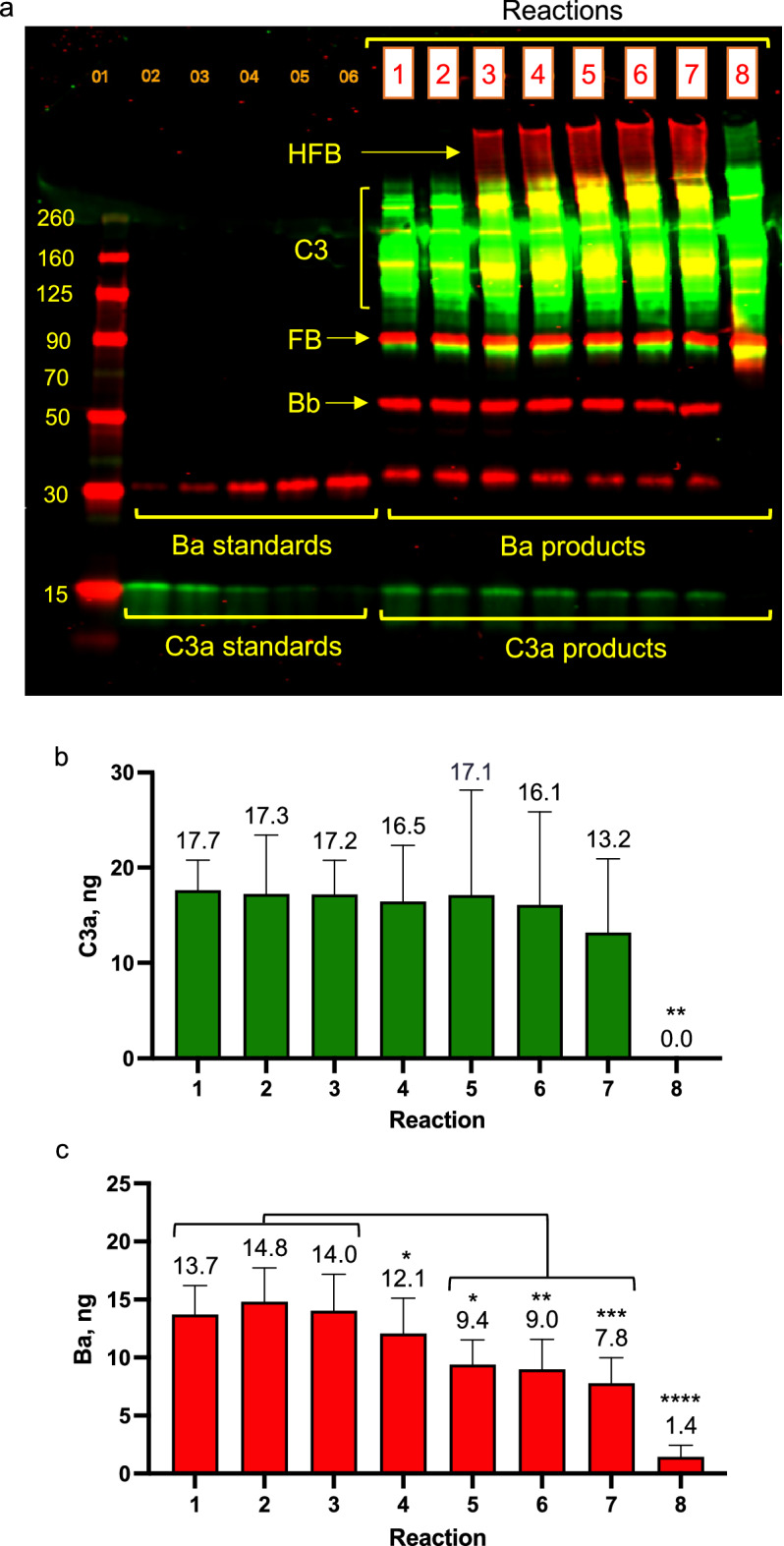
HFB inhibition of AP activation in FH-D serum (with low FH). AP activation was measured by Western blot analysis in FH-D serum (supplemented with 3.13 ng/µl of FH) after the addition of increasing concentrations of HFB. FH was not added to FH-D serum in control reactions 1, 7 and 8. (**a**) The representative Western blot shown is the merged detection of C3a (using rabbit anti-C3a + donkey anti-rabbit IRDye-800, green) and Ba (using goat anti-FB + donkey anti-goat IRDye-680, red). The protein ladder is in lane 1, C3a and Ba standards are in lanes 2–6, and the reaction samples (labeled with boxed numbers 1–8) are in lanes 8–14. The extent of AP activation was measured after 5 min at 22 °C and analyzed for (**b**) C3a and for (**c**) Ba, in 4 experiments. In (**a**), C3a levels measured in the absence of FD were significantly lower than levels measured in the other reactions (1 vs. 8, P = 0.0016; 2 vs. 8, P = 0.0025; 3 vs. 8, P = 0.0021; 4 vs. 8, P = 0.0033; 5 vs. 8, P = 0.0022; 6 vs. 8, P = 0.004; 7 vs. 8, P = 0.0246). In (**b**), Ba levels in reactions 5, 6 and 7 were statistically lower than Ba levels in reactions 1–3; (1 vs. 5, P = 0.0154; 1 vs. 6, P = 0.007; 1 vs. 7, P = 0.0006; 2 vs. 5, P = 0.0016; 2 vs. 6, P = 0.0008; 2 vs. 7, P < 0.0001; 3 vs. 5, P = 0.0079; 3 vs. 6, P = 0.0035; 3 vs. 7, P = 0.0003) and levels in reaction 7 were statistically lower than reaction 4 levels (P = 0.0163). In the absence of FD, the measured levels of Ba were lower than levels in each of the other reactions (1–6 vs. 8, P < 0.0001; 7 vs. 8, P = 0.0003). Statistics were analyzed by 2-way ANOVA with Tukey’s multiple comparisons test and were summarized on graph (*P < 0.05, **P < 0.005, ***P < 0.0005, ****P < 0.0001).

Increasing concentrations of HFB were added to FH-D serum supplemented with 3.13 ng/µl FH (12.5% of normal serum levels). HFB concentrations were 2-, 2.5-, 3-, and 3.5-fold above reaction FB levels of 10.4 ng/µl (Table 3). Levels of Ba, but not C3a, were reduced with increasing concentrations of HFB. C3a levels in reactions with the highest concentration of HFB were only incrementally lower than C3a levels in reactions with FH-D serum alone (without added FH, reaction 1, Fig. 3b). In contrast, Ba levels in reactions with additions of HFB at 3- and 3.5-fold higher than reaction FB concentrations, were statistically lower than Ba produced in: FH-D serum alone; FH-D serum plus low FH (without HFB addition); and FH-D serum plus low FH containing the lowest concentration of HFB tested (Fig. 3c). Ba levels in FH-D serum plus low FH (reaction 2) were reduced by 37% with 31.2 ng/µl HFB (reaction 5), and by 40% with 36.4 ng/µl HFB (reaction 6). Similar reductions in Ba generation (47% lower) were measured in FH-D serum containing 36.4 ng/µl HFB, without the addition of FH (reaction 7) (Fig. 3c). Negligible amounts of C3a and Ba were produced in FH-D serum reactions without added Mg^+2^ ions (or Ca^+2^ ions), further verifying that the C3a and Ba generated in FH-D serum resulted from AP activation (Fig. 3b,c).

**Table 3:** FH and HFB concentrations in FH-D serum reactions.

Reaction description	Reaction conc., ng/µl		HFB to FB ratio
	FH	HFB	
1. FH-D serum alone	0	0	–
2. FH-D serum, 3.13 FH	3.13	0	–
3. FH-D serum, 3.13 FH, 20.8 HFB	3.13	20.8	2-fold
4. FH-D serum, 3.13 FH, 26 HFB	3.13	26	2.5-fold
5. FH-D serum, 3.13 FH, 31.2 HFB	3.13	31.2	3-fold
6. FH-D serum, 3.13 FH, 36.4 HFB	3.13	36.4	3.5-fold
7. FH-D serum, 36.4 HFB	0	36.4	3.5-fold
8. FH-D serum, without Mg^+2^ ions	0	0	–

Protein concentrations of FH and HFB in reactions containing FH-D serum, low FH and increasing amounts of HFB. In reactions, the FH-D serum was diluted in 0.1 mM EDTA to produce final concentrations of 60 ng/µl C3 and 10.4 ng/µl FB. The FH concentration was equivalent to 12.5% of normal serum levels. C3a and Ba levels were measured after 5 min at 22 °C. The results are in Fig. 3. The values in the Reaction description are protein concentrations in ng/µl.

### AP activation on EC-secreted ULVWF strings

#### Endothelial cell stimulation and endogenous complement protein release

Fluorescent microscopy was used to measure quantities of endogenously released complement proteins attached to EC-secreted ULVWF strings in the presence or absence of HFB. Human glomerular microvascular endothelial cells (GMVECs) were stimulated with histamine and ULVWF strings were secreted from Weibel-Palade bodies rapidly (within 2 min) onto cell surfaces. The antibodies added to detect VWF (15 min) also impaired EC-released ADAMTS-13 from cleaving the surface ULVWF strings. Additionally, the 15 min period allowed endogenous complement proteins to be released in sufficient amounts to measure their attachment to the ULVWF strings. Subsequently, the GMVECs were fixed and antibodies were added to detect C3, FB, C5 and C4 proteins.

#### Verification of EC-released complement protein attachment to the ULVWF strings

**Figure 4:**
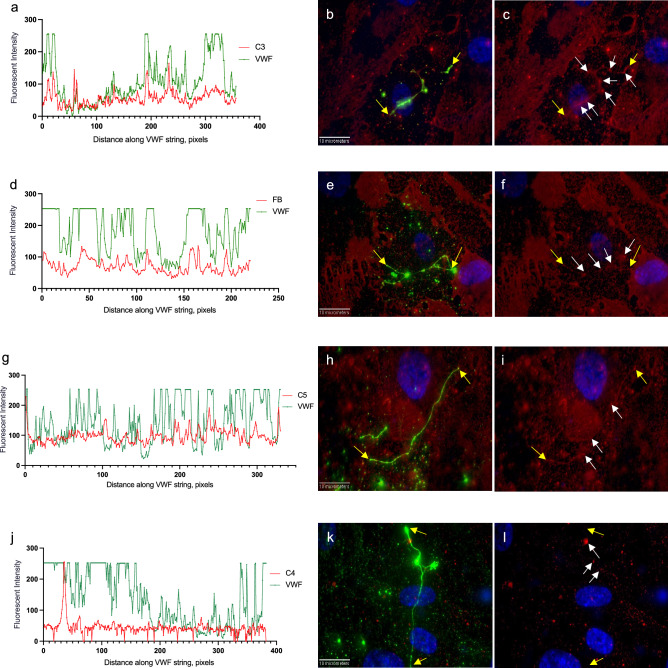
GMVEC-released C3, FB, C5, although not C4, bind to cell-secreted ULVWF strings. GMVECs were stimulated with 100 µM histamine for 2 min, followed by addition of rabbit anti-VWF + chicken anti-rabbit AF-488 (green) for 15 min. Cells were then p-formaldehyde-fixed and stained with goat antibodies against: C3 (**a**–**c**); FB (**d–f**); C5 (**g–i**); and C4 (**j–l**); and secondary donkey anti-goat AF-647 (red). Cell nuclei were detected with DAPI (blue). Graphs show the fluorescent intensities (y-axis) measured along the ULVWF string (x-axis, length in pixels, 1 pixel = 0.114 µm) from merged images of detected VWF with each complement protein. The yellow arrows in the merged images indicate the start and end points of the strings. The white arrows in the merged images of complement proteins (647 nm, red) with DAPI detection indicate high 647-intensity values (in order to assist visualization of the measured line). Images were obtained at 60X and were selected from over 100 images.

In order to substantiate that GMVEC-released complement proteins attached to the ULVWF strings, detected intensities for C3, FB, C5 and C4, were measured along a single GMVEC-secreted string. Graphs of the measured intensities versus the length of the ULVWF string were generated. Each complement protein (Fig. 4a,d,g), except C4 (Fig. 4j), showed patterns of synchronized detection with VWF. Background string intensities for each protein were measured from identical line shapes moved to another location within the same image (Supplementary Fig. S5). The attachment intensities, background intensities, and the ULVWF string lengths, measured from images in Fig. 4 are in Supplementary Table S2.

**Figure 5:**
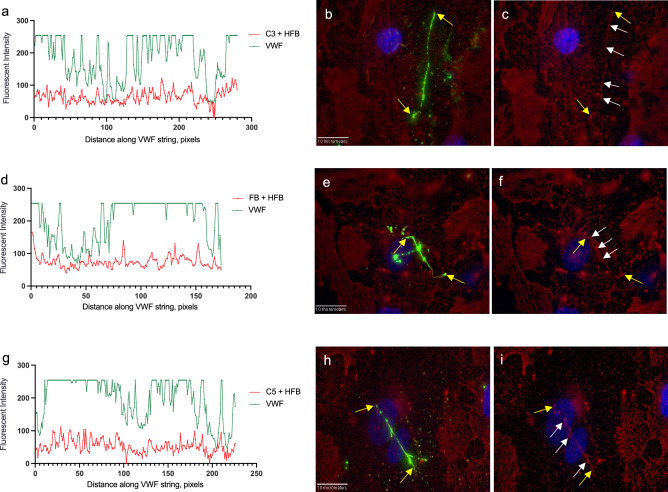
C3, FB and C5, even in the presence of HFB, bind to GMVEC-secreted ULVWF strings. GMVECs were stimulated with 100 µM histamine containing 20 µg/ml HFB for 2 min, followed by addition of rabbit anti-VWF + chicken anti-rabbit AF-488 (green) for 15 min (also in the presence of HFB). Cells were then p-formaldehyde-fixed and stained with: goat anti-C3 (**a–c**); goat anti-FB (**d–f**); or goat anti-C5 (**g–i**); and secondary donkey anti-goat IgG AF-647 (red). Cell nuclei were detected with DAPI (blue). Graphs show the fluorescent intensities (y-axis) measured along the ULVWF string (x-axis, length in pixels, 1 pixel = 0.114 µm) from merged images of detected VWF with detected C3, FB, or C5 (with HFB present during incubation). The yellow arrows in the merged images indicate the start and end points of the strings. The white arrows in the merged images of complement proteins (647 nm, red) with DAPI detection indicate high 647-intensity values (in order to assist visualization of the measured line). Images were obtained at 60X and were selected from over 130 images.

In parallel experiments, GMVECs were both histamine-stimulated and stained with VWF antibodies, in the presence of HFB. Detected signals for C3, FB and C5 were also synchronized with VWF detection along the string with the addition of HFB (Fig. 5a,d,g). Background string locations and graphs for each protein measured in the presence of HFB are in Supplementary Fig. S6. Additional data measured from the images in Fig. 5 are included in Supplementary Table S2.

#### C3, FB and C5, but not C4, attached to GMVEC-secreted ULVWF strings

**Figure 6:**
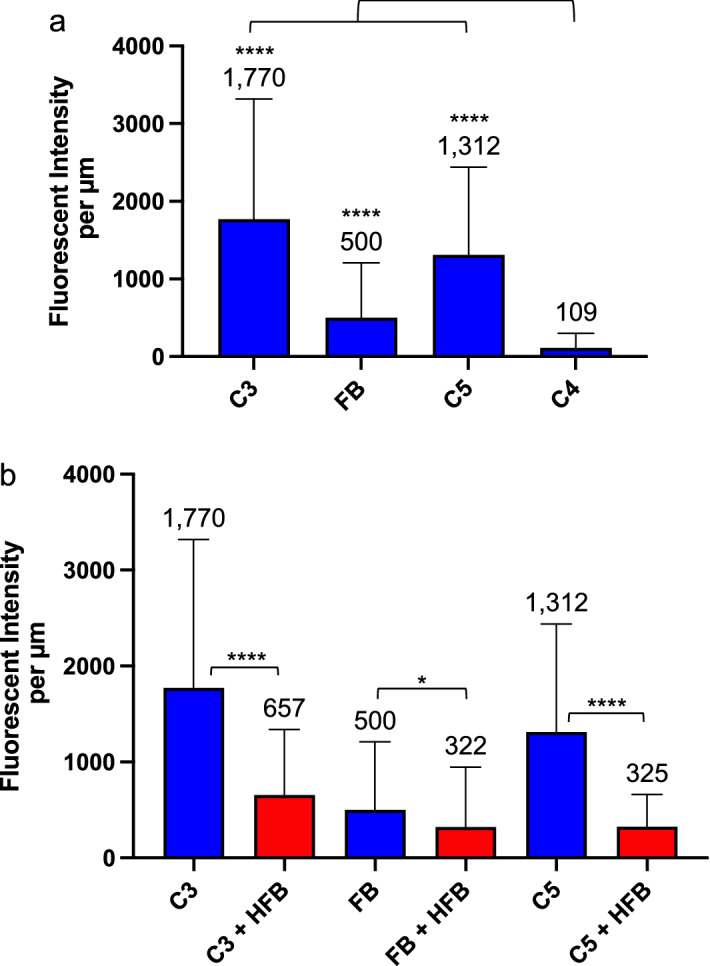
Quantification of GMVEC-released complement proteins attached to GMVEC-secreted ULVWF strings in the presence or absence of HFB. Intensities of GMVEC-released complement proteins were measured along ULVWF strings secreted in response to: (**a**) 100 µM histamine; and (**b**) histamine ± 20 µg/ml HFB. Shown are the complement component fluorescent intensities per micron of ULVWF string length after background string subtraction. Values are means plus SD; n = 65–151 strings for proteins without HFB (blue bars) and 151–320 strings for proteins with HFB (red bars). Data were compiled from 3 to 9 experiments per condition and over 800 fluorescent images. In (**a**), values for C3, FB and C5 are statically different from C4 (****P < 0.0001); and in (**b**), paired comparison values for C3 and C3 + HFB (****P < 0.0001), C5 and C5 + HFB (****P < 0.0001) and FB and FB + HFB (*P = 0.0311) are statistically different by one-way ANOVA, non-parametric analysis.

C3 and C4 bind to surfaces, as C3b and C4b, through thioester groups exposed after cleavage/activation^1,36,37^. However, non-activated forms of FB and C5 initially bind to C3b within C3/C5 convertases before cleavage to Bb and C5b. The amount of complement protein-attachment to the ULVWF strings was calculated as the sum of fluorescent intensity measured along the strings divided by the length (in microns) of the ULVWF strings. These intensity data were compiled from over 800 microscope images. Mean intensity values measured for C3 (1770/µm), C5 (1312/µm), and FB (500/µm) attachment to the ULVWF strings were 16-fold, 12-fold, and 4.5-fold higher than mean intensity values for C4 (109/µm) attachment (Fig. 6a). For each FB protein attached to the ULVWF strings, there were 3.5 components of C3 and 2.6 components of C5. These attachment ratios are compatible with the formation of C3 and C5 convertases on the ULVWF strings.

#### HFB decreased C3, FB and C5 attachment to ULVWF strings

In analogous experiments, the ULVWF-attachment of C3 (as C3b), FB and C5 was measured with HFB (20 µg/ml) present during histamine stimulation and VWF antibody incubation. FB, after heat-inactivation at 56 °C, retains binding capacity to C3b, although HFB cleavage by FD is either reduced or absent. (In Fig. 1, reaction 2 consisting of C3, FD and HFB, neither C3a nor Ba was generated.) HFB may compete with GMVEC-released endogenous FB for binding to fluid phase C3-H_2_O. This would likely limit C3b attachment to the ULVWF strings and, consequently, reduce further amplification of C3b formation. HFB may also compete with endogenous FB for binding to C3b that is bound to ULVWF strings, under conditions where GMVEC-released FH has displaced either FB or Bb^10,38^.

The presence of HFB reduced C3 attachment by 2.7-fold (657/µm), FB attachment by 1.5-fold (322/µm) and C5 attachment by fourfold (325/µm). In the presence of HFB, for each FB or HFB attached to the ULVWF strings, there were 2 components of C3 and 1 component of C5. These are the proportions of the C5 convertase complex after C5 binding [(C3bBbC3b)-C5] (Fig. 6b). Amounts of each protein attached to ULVWF strings, in the presence of HFB, were statistically different from ULVWF-attached protein amounts in the absence of HFB. The most prominent effect of HFB was to reduce the amount of C5 attachment to the ULVWF strings. C5 preferably binds to C3bBb attached to, or adjacent to, additional surface-bound C3b^5^. The reduced number of ULVWF-bound C3b in the presence of HFB, along with the formation of C3 convertases comprised of HFB instead of Bb, may contribute to the decrease in C5 attachment to the ULVWF strings (Fig. 6b).

## Discussion

In this study, Western blot analysis was used to measure the effect of HFB in reducing AP activation by quantifying activation products, C3a and Ba, in reactions using purified AP proteins or FH-D serum. This analytical technique enabled us to quantify Ba in the presence of high levels of HFB that interfere with Ba measurement in immunoassays; and measure C3a levels more precisely than by immunoassays. C3a levels were more accurately measured in reaction samples prepared for Western blot analysis, because AP activation was instantly stopped, and further activation was prevented, by the addition of protein-denaturing buffer (containing EDTA) and rapid heating. In typical immunoassays, measurement samples are exposed for extended periods to mild conditions (in order to promote antibody binding and substrate detection). These latter conditions also allow further activation. An excess of EDTA added to samples prevents further convertase formation; however existing convertases continue to cleave C3 and generate C3a^38^.

The usefulness of HFB in reducing over-activation of the AP was evaluated in this study. Initial reactions, with constant C3 and FD levels and varying FB concentrations, were used to test the possibility that AP activation could be reduced by lowering FB levels. The addition of HFB effectively lowered the FB concentration because the two proteins compete for binding to C3b. Heat-inactivated FB was prepared from FB that had been purified from human plasma and then exposed to controlled heating at 56 °C. HFB was determined to be inactivated if activation products were not generated in reactions consisting of C3, HFB and FD, without the addition of FB (Fig. 1a–c, reaction 2). (FB is the only heat-labile AP enzyme in the activation pathway^39^). HFB effectively competed with native FB for binding to C3b. Because of heat-induced impaired enzymatic functions, HFB-C3b complexes were unable to promote further activation. (The structural changes that occur in FB after heating have not been thoroughly investigated^40^). On our Western blots, the migration pattern of HFB indicated that it is composed of dimers and tetramers of FB monomers (93 kDa). Clear bands of HFB are shown in the blot detected for FB in Supplemental Fig. S3. In the lane of reaction 2, (HFB with purified proteins, C3 and FD), there are two prominent bands for HFB plus some HFB remaining in the well. The lower HFB band, at 186 kDa, is likely a FB dimer, whereas the upper band may be a FB tetramer (~ 372 kDa).

In plasma, C3 is continually hydrolyzed to a C3b-like configuration, C3(H_2_O) that allows FB binding and C3 convertase formation. In the fluid phase, FH prevents any unwanted C3 convertase formation by displacing either FB or Bb that is bound to C3b or to C3(H_2_O). In the absence of FH, the AP in human serum is activated spontaneously and precipitately upon the addition of mM levels of magnesium ions^4^. Because of this unique property, FH-D serum was used as a reagent to test the effectiveness of HFB in inhibiting AP activation in the presence of the full spectrum of proteins in normal serum (including complement regulators), with the exception of FH (until experimental addition). This addition of FH (to 12.5% normal level) brought the FH-D serum within the range of FH (5–50% of normal) observed in many patients with aHUS resulting from heterozygous mutations in FH or FH autoantibodies^15,16,19,29^. FH-D serum was initially shown to be devoid of FH by blot analysis using samples with twofold the serum amounts that were used in the FH-D serum AP reactions (Supplementary Fig. S4).

In the absence of HFB, AP activation was mostly higher in the FH-D serum-containing reactions than in reactions using purified AP proteins. Levels of C3a (10 ng/ml) and Ba (9 ng/ml), generated in reactions of purified AP proteins, were nearly half the levels produced in FH-D serum upon addition of Mg^+2^ ions (18 ng/ml and 14 ng/ml, respectively). Although basal levels of C3a and Ba in the FH-D serum preparations contributed slightly, the increased activation probably resulted from the capacity of properdin to bind and stabilize C3/C5 convertases. The short (2–3 min) half-life of C3bBb can be increased tenfold with properdin binding^41,42^.

Negligible amounts of C3a and Ba were produced in FH-D serum reactions in EGTA buffer without added Mg^+2^ ions (or Ca^+2^ ions) (Fig. 3, reaction 8). Activation of C3, producing C3a, also occurs in the classical and lectin pathways, providing that Mg^+2^ ions (for C2 binding to C4b) and Ca^+2^ ions (for C1 complex formation) are present. In contrast, AP activation, specifically FB binding to C3b, requires only Mg^+2^ ions^38^.

In the reactions using FH-D serum (supplemented with low FH levels), HFB reduced Ba generation, whereas C3a generation was mostly unchanged. In the fluid phase and in the absence or insufficient levels of regulatory proteins, C3 convertases (C3bBb), rapidly cleave C3, producing C3b and releasing C3a. HFB competes with native FB for binding sites on newly formed C3b. We propose that HFB is resistant to cleavage by FD, and therefore, C3b-HFB complexes do not produce Bb or generate Ba. Consequently, the C3b-HFB complexes would be incapable of further C3 cleavage; however, C3a would continue to be generated from the previously formed C3 convertases.

The effectiveness of HFB in limiting AP convertase formation on activated surfaces was evaluated using stimulated human GMVECs. ECs produce complement components and the associated fluid-phase^7,10^ and surface regulatory proteins necessary to control complement over-activation^6–12^. EC surface proteins that function to regulate activation include: CD55 (to displace the enzyme subunits of convertases formed on its membrane); CD59 (to prevent polymerization and attachment of C9 into C5b-8 complexes); as well as, CD46 and CD141 (to inactivate cell membrane-bound C3b in association with co-factors FH and FI)^43^. CD141, also known as thrombomodulin, is present exclusively on EC surfaces and participates substantially in controlling coagulation^44^.

Fluorescent microscopy was used to analyze and quantify the attachment of complement proteins to the GMVEC-secreted ULVWF strings. The polyclonal antibodies made against human proteins were shown previously to identify each specific complement component and their activated forms^12^. The GMVEC-secreted ULVWF strings remained on EC surfaces because the added detection antibodies against VWF impeded string cleavage by ADAMTS-13 released from ECs^12^.

The endogenously released AP components, C3, FB and C5, were bound to the ULVWF strings in numerical ratios consistent with the formation of active C3 convertases (C3bBb) and C5 convertases (C3bBbC3b). There was minimal binding of the classical component C4 to the ULVWF strings, indicating little activation of the classical and lectin pathways. The presence of HFB reduced the binding of C3 (as C3b) and C5 to the ULVWF strings by 2.5-fold and fourfold, respectively. Heat-inactivated FB is resistant to FD cleavage; however, HFB retains binding capacity for C3b. The HFB competes with endogenous FB for binding to C3b and suppresses further cleavage of C3 to activated C3b. This resulted in reduced C3 and C5 convertase assembly on the GMVEC-secreted ULVWF strings.

The addition of HFB reduced C5 binding to the ULVWF strings by fourfold, probably as a result of the reduced amount of C3b-ULVWF attachment (2.5-fold lower) in the presence of HFB. Surfaces with high densities of C3b deposition have an increased affinity for C5 binding^5^. Reduced C5 binding, translates into: a decreased number of C5 convertases; fewer C5b-6 complexes (precursors to lytic membrane attack complexes, C5b-9); and reduced amounts of generated C5a. C5a is a potent anaphylatoxin, and among other biological properties, induces the release of histamine and tumor necrosis factor, potentiating inflammation and cell injury^45–47^.

HFB inhibits an early reaction of AP activation and, therefore, may be of therapeutic value in preventing AP-mediated generation of C3a and C5a. This inhibitory function may help to suppress the associated inflammation caused by an overactive AP in aHUS and related disorders.

## Materials and methods

### AP activation in fluid-phase reactions

#### Validation of linear detection using Western blot analyses

C3 (A113) and C3a-desArg (A119) proteins, ranging from 7.5 to 240 ng/lane, were analyzed by Western blotting under reducing conditions. Membranes were initially stained with Revert 700 Total Protein Stain (LI-COR, 926-11010) and subsequently incubated with rabbit anti-human C3a (A218) plus donkey anti-rabbit IRDye-800CW (1:20,000, LI-COR, 926-32213) for specific detection of C3 and C3a. Parallel experiments, using non-reduced FB (A135) and Ba (A154) proteins ranging from 7.5 to 240 ng/lane, were also analyzed by Western blotting, stained for total protein, followed by specific detection with goat anti-human FB (A235) plus donkey anti-goat IRDye-800CW (1:20,000, LI-COR, 926-32214). Complement proteins and antibodies were purchased from Complement Technology.

#### The extent of AP activation is FB-dependent

AP activation experiments were conducted on ice using pre-cooled reagents. C3 aliquots were only used once. AP proteins C3, FB, and FD (A136, Complement Technology) were diluted in Tris/EGTA-Mg buffer (20 mM Tris, pH 7.4, 5 mM EGTA, 5 mM MgCl_2_). C3 was the last protein added to the 25 µl reaction mixtures. Reaction concentrations of C3 (60 ng/µl) and FD (0.07 ng/µl) remained constant (at normal serum ratios), whereas FB concentrations were 20, 10 and 5 ng/µl. After C3 addition, the tubes were mixed and incubated at 37 °C for 15 min. Reactions were stopped by the addition of 5 mM EDTA and 25 µl of Laemmli sample buffer (BioRad, 161-0737), followed by heating at 95 °C for 5 min. AP proteins were measured by fluorescent blot analysis using rabbit anti-C3a + donkey anti-rabbit IRDye-800 and goat anti-FB + donkey anti-goat IRDye-680.

#### Heat-inactivated factor B^34^

(HFB) was prepared from human plasma-purified FB (A135, Complement Technology), inactivated by controlled heating at 56 °C for 30 min using a thermocycler and stored at 4 °C until use.

#### Heat-inactivated FB (HFB) inhibits activation of the AP

AP activation experiments were conducted on ice using pre-cooled reagents. C3 aliquots were only used once. AP proteins C3, FB, FD and HFB were diluted in Tris/EGTA-Mg buffer (20 mM Tris, pH 7.4, 5 mM EGTA, 5 mM MgCl_2_). C3 was the last protein added to the 25 µl reaction mixtures. Final reaction concentrations were: C3 = 60 ng/µl; FB = 10.4 ng/µl; and FD = 0.07 ng/µl. Final concentrations of HFB were: 10.4, 15.6, 20.8, 26, and 31.2 ng/µl. After C3 addition, the tubes were mixed and incubated at 37 °C for 15 min. Reactions were stopped by the addition of 5 mM EDTA and 25 µl of Laemmli sample buffer (BioRad,161–0737), followed by heating at 95 °C for 5 min. AP proteins were measured by fluorescent blot analysis with standard curves for C3a and Ba.

#### Fluorescent Western blot analysis

Proteins were separated by 4–15% Tris–Glycine SDS-PAGE (BioRad, 456-1086) under non-reducing conditions. Gels included standard lanes of C3a-desArg (32–1.25 ng/lane) and Ba (20–2.5 ng/lane) proteins for quantification and a pre-stained protein ladder (LI-COR, 928-6000) for approximating size. Gels were transferred using a semi-dry method (BioRad Trans-Blot, 170-3940) onto low-fluorescence PVDF membranes (IPFL 10100). Remaining steps followed protocols recommended by LI-COR using TBS-based Intercept Blocking Buffer (LI-COR, 927-00001) for membrane blocking and antibody dilution. Primary antibodies were diluted in Intercept buffer containing 0.2% Tween-20 and diluent for fluorescent secondary antibodies contained 0.2% Tween-20 plus 0.01% SDS. Ba protein was detected using goat anti-human FB (4.3 µg/ml) plus donkey anti-goat IRDye-680RD (1:20,000, LI-COR, 926-68074). C3a protein was detected using rabbit anti-human C3a (6.9 µg/ml) plus donkey anti-rabbit IRDye-800CW (1:20,000, LI-COR, 926-32213). Dried membranes were scanned on a LI-COR Odyssey DLx Imager (9142-01P), intensities were measured at 700 and 800 nm, and data were analyzed using Empiria Studio software (LI-COR, 2000-000).

### AP activation in Factor H-depleted (FH-D) serum

#### Human Factor H-depleted serum

Factor H-depleted serum (FH-D serum; A337, Complement Technology) was prepared from human serum by immuno-depletion of FH. FH-D is supplied in 0.1 mM EDTA and contains normal levels of each complement protein except FH. C3 levels in FH-D serum were estimated at 1200 µg/ml, based on the supplier statement that FH levels of 500 µg/ml is required for 100% AP function, and that in normal serum C3 levels are 2.4-fold higher than FH levels. In the reactions, FH-D serum was further diluted in 0.1 mM EDTA, in order to produce C3 concentrations of 60 ng/µl and 10.4 ng/µl FB. The reactions were supplemented with EGTA/MgCl_2_ buffer to provide final concentrations of 5 mM MgCl_2_.

#### FH levels in FH-D serum

FH levels in FH-D serum were measured by fluorescent Western blot analysis. FH-D serum was diluted 20-. 30-, 40-, 60-, 80-, and 120-fold directly into Laemmli sample buffer containing 5 mM EDTA. FH protein (A137, Complement Technology) standards in gel lanes ranged from 15, 30, 45, 60, 75, 90 and 120 ng. Blots were detected for FH using goat anti-FH (A237, Complement Technology) plus donkey anti-goat IRDye-680 and for the serum-containing protein, vitronectin, with rabbit anti-vitronectin (A260, Complement Technology) plus donkey anti-rabbit IRDye-800CW.

#### AP activation measured in FH-D serum with FH addition

Single-use aliquots of FH-D serum were diluted 1:4.8 in 0.1 mM EDTA to produce C3 levels of 250 ng/µl. To initiate AP activation, a constant volume of diluted FH-D serum was added to tubes containing Tris/EGTA-Mg buffer alone, or dilutions of FH protein. Reactions contained 60 ng/µl of C3 and 10.4 ng/µl FB in a total volume of 25 µl. FH protein, diluted in Tris/EGTA-Mg buffer, ranged from 0.78 to 25 ng/µl to produce FH levels equivalent to 3–100% of normal serum levels. Reaction mixtures were incubated for 15 min at 37 °C. The reactions were stopped by the addition of 5 mM EDTA in Laemmli sample buffer and heating at 95 °C for 5 min. Levels of AP activation proteins, C3a and Ba, were measured by fluorescent blot analysis.

#### AP activation in FH-D serum with HFB addition

FH-D serum was supplemented with 3.13 ng/µl FH (12.5% of normal serum levels) to study the effect of HFB on AP activation in FH-D serum. This chosen concentration of FH allowed enough generation of C3a and Ba to assure that C3a and Ba levels would remain within measurable ranges during HFB inhibition of AP activation. Additions of ≥ 12.5 ng/µl FH were found to substantially prevent AP activation (Fig. 2). FH-D serum was diluted in 0.1 mM EDTA to produce C3 levels of 250 ng/µl. To initiate AP activation, a constant volume of diluted FH-D serum was added to tubes containing Tris/EGTA-Mg buffer alone, or to tubes containing 3.13 ng/µl FH and increasing concentrations of HFB. Reaction HFB concentrations were 20.8, 26, 31.2, and 36.4 ng/µl representing levels 2-, 2.5-, 3-, and 3.5-fold higher than reaction FB serum levels of 10.4 ng/µl. Reaction mixtures were incubated for 5 min at room temperature before the reactions were stopped by the addition of 5 mM EDTA in Laemmli sample buffer and heating at 95 °C for 5 min. Levels of AP activation proteins, C3a and Ba, were measured by fluorescent blot analysis.

### AP activation on EC-secreted ULVWF strings

#### Human glomerular microvascular endothelial cells (GMVECs).

Primary GMVECs (Cell Systems, ACBRI-128 V, single donor at passage 2), were grown to confluence on gelatin-coated coverslips in MCDB-131 basal medium plus 120 units/ml penicillin; 100 µg/ml streptomycin; 2 mM L-glutamine; 0.25 µg/ml amphotericin and microvascular growth supplement (S00525, with 5% v/v FBS, Life Technologies). GMVECs, used at passages 4–7, were removed from tissue culture flasks non-enzymatically, prior to seeding on coverslips, by incubation with 5 mM EDTA in Ca^+2^, Mg^+2^-free PBS and gentle cell scraping.

#### Microscope image acquisition.

Our microscope system consists of a Nikon Diaphot TE300 microscope equipped with CFI Plan Fluor 60 × oil, numerical aperture (NA) 1.4 and CFI Plan Apo Lambda 100 × oil, NA 1.45 objectives, a 10 × projection lens and a Prior motorized stage. Fluorescent images were obtained with a SensiCamQE CCD camera (Cooke) using dual filter wheels (Prior) with single band excitation and emission filters for FITC/TRITC/CY5/DAPI (Chroma). Cell images were captured using IP Lab software version 3.9.4r4 (Scanalytics) and processed using iVision-Mac Scientific Image Processing version 4.5.5r1 (Biovis.com). Images acquired using the 60 × objective have dimensions of 78 × 58 µm. Calibration bars on images are 10 µm.

#### GMVEC stimulation, HFB incubation and fluorescent staining

GMVECs on coverslips (7–15 days after seeding) were washed with PBS and stimulated with 100 µM histamine in 1 ml PBS with or without 20 µg/ml HFB for 2 min. Cells were then stained with rabbit anti-VWF plus chicken anti-rabbit Alexa Fluor (AF)-488 (A-21441, Invitrogen) for 15 min, washed with PBS and fixed with 1% p-formaldehyde/PBS for 10 min. Following fixation, the cells were stained with one of the polyclonal goat antibodies against human complement; anti-C3 (A213), anti-C4 (A205), anti-C5 (A220), and anti-FB (A235) (Complement Technology) (diluted 1:100 in PBS containing 1% BSA) plus secondary donkey anti-goat AF-647 (A-21447, Invitrogen) antibody at 20 µg/ml for 15 min. These two fluorophore dyes, AF-488 and AF-647, have widely separated, non-overlapping, absorption and emission spectra. Cell nuclei were detected with DAPI (4’,6-diamidino-2-phenylindole, 1.5 µg/ml) that was included in the mounting medium.

#### Intensity measurements of complement proteins on ULVWF strings

The GMVEC-secreted ULVWF strings, detected with rabbit anti-VWF plus chicken anti-rabbit AF-488, were electronically traced as lines in 488 nm (green)-captured images at 60X. The ULVWF string length (major axis) and integrated fluorescent intensity was measured along the line. The x- and y-coordinates of the traced ULVWF line were transferred to the corresponding 647 nm (red)-captured images obtained using goat antibodies against C3, FB, C4 and C4 plus donkey anti-goat AF-647. The fluorescent intensity at 647 nm from each detected complement component was measured and integrated at the transferred line coordinates. Background intensities were measured from identical line coordinates translocated from original positions within the same 647-detected image. The quantity of each complement component attached to the ULVWF strings was expressed as complement component intensity at 647 nm, minus the background intensity at 647 nm, divided by the ULVWF string length in microns. Single channel images have maximum intensity values of 4096. Image dimensions at 60 × are 78 µm × 58 µm, or 688 pixels × 512 pixels, (1 pixel = 0.114 µm).

#### Intensity plots of complement proteins attached to individual ULVWF strings

Intensities from 488 nm (VWF, green) and 647 nm (complement proteins, red) channels were measured along the ULVWF strings from merged images. The x-axis is the length of the VWF string in pixels (100 pixels = 11.4 µm) and the y-axis is the measured intensity. Multiple-channel merged images have maximum values of 256. The background 647 nm data was measured from the same merged image where the x- and y-pixel coordinates for the ULVWF string had been relocated to another position within the image.

#### Statistical analysis

GraphPad Prism v 9.4.1 software (GraphPad.com) was used for standard curve interpolation, Pearson’s correlation coefficient measurements, and for determining significance of differences using 1-way ANOVA and Dunnett’s multiple comparison tests, and 2-way ANAOVA with Tukey’s multiple comparison with an alpha value of 0.05.

## Supplementary Information

Supplementary Information.

## Data Availability

The datasets generated during and/or analyzed during the current study are available from the corresponding author on reasonable request.
