# Supplementary material for: Heat-inactivated Factor B inhibits alternative pathway fluid-phase activation and convertase formation on endothelial cell-secreted ultra-large von Willebrand factor strings
Source: Sci Rep. 2023 Apr 8;13:5764. doi: 10.1038/s41598-023-33007-3 (PMC10082794; doi:10.1038/s41598-023-33007-3)
Supplement: Supplementary file 1 — Supplementary Information. [file 41598_2023_33007_MOESM1_ESM.pdf]

Heat-inactivated Factor B inhibits alternative pathway fluid-phase activation and convertase formation on endothelial cell-secreted ultra-large von Willebrand factor strings

Nancy A. Turner*, Joel L. Moake

Supplementary Material

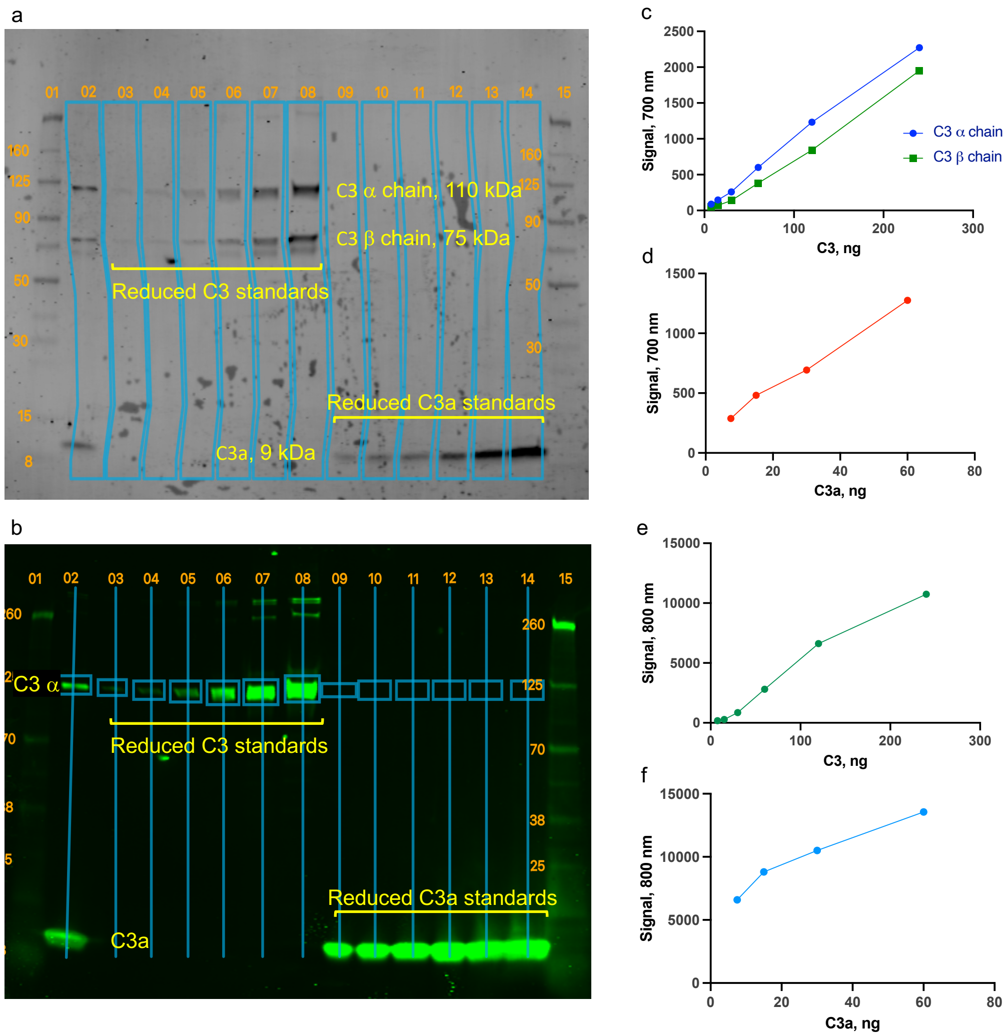

Supplementary Fig. S1, a-f. Validation of C3 and C3a protein amounts used in Western blot analyses. Complement proteins C3 and C3a-desArg (C3a), ranging from 7.5 to 240 ng/lane, were analyzed on Western blots for total protein and by specific antibody detection. Protein ladders are in lanes 1 and 15. (**a** and **b**) Reduced samples of C3 (lanes 3-8) and C3a (lanes 9-14) were detected in (**a**) by total protein stain (entire lane intensity) at 700 nm (grayscale); and in (**b**) with rabbit anti-human C3a plus donkey anti-rabbit IRDye-800 (specific band intensity) at 800 nm (green). The C3a antibody does not detect the C3 beta chain. Lane 2 contains 30 ng of C3 and of C3a. (**c**, **d**) Signal intensity plots of total lane protein for: (**c**) C3 alpha chain (r^2 = 0.9976) and C3 beta chain (r^2 = 0.9948); and (**d**) C3a (r^2 = 0.9955, ≤ 60 ng) versus lane amounts of C3 and C3a proteins. (**e**, **f**) Signal intensity plots of band intensities detected by C3a antibody for: (**e**) C3 (r^2 = 0.9999); and (**f**) C3a (r^2 = 0.9584, ≤ 60 ng) versus lane amounts of C3 and C3a proteins. Coefficients of determination (r^2) were calculated using simple linear regression and in (**e**) using a non-linear four-parameter logistic curve fit.

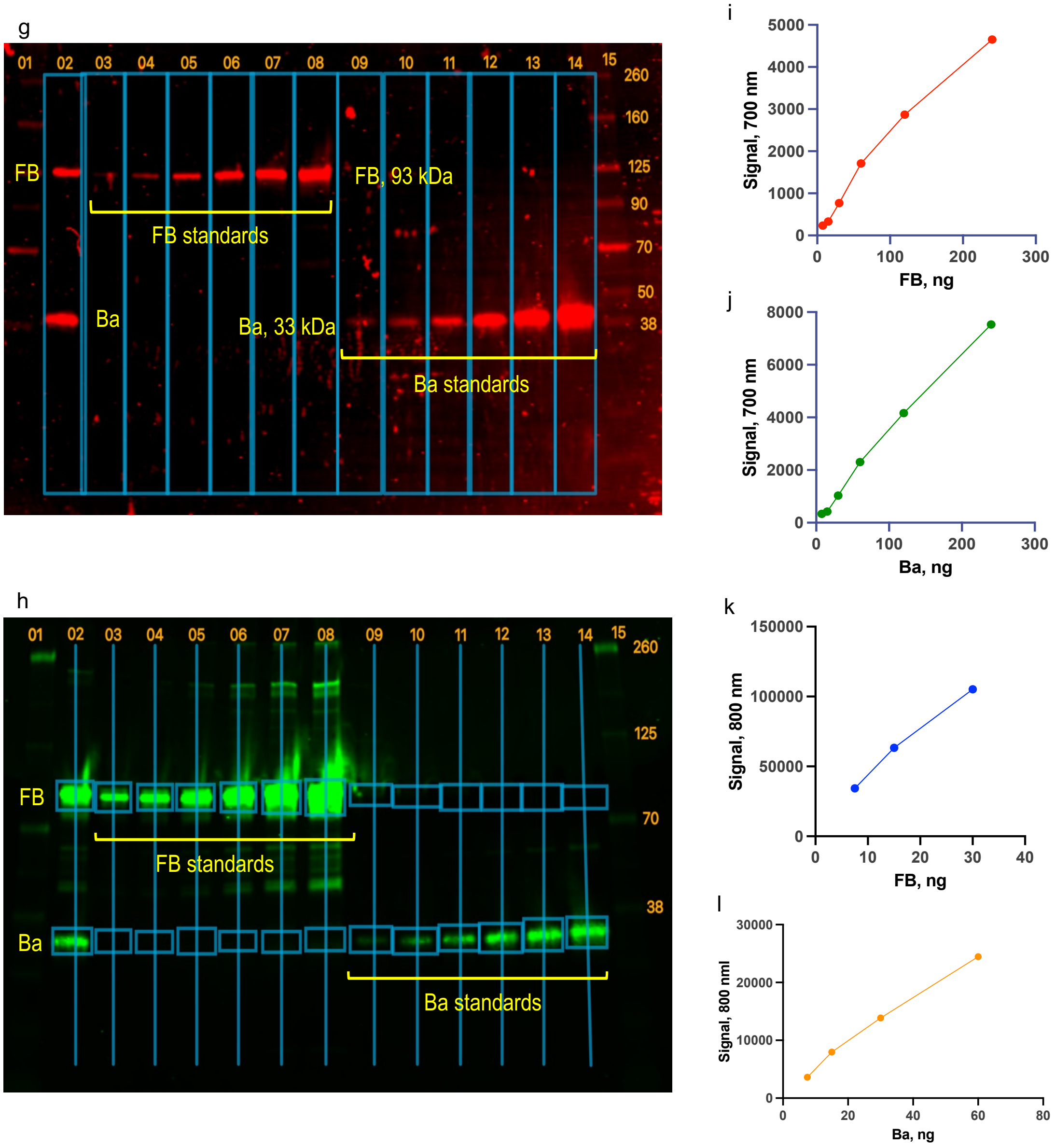

Supplementary Fig. S1, g-l. Validation of FB and Ba protein amounts used in Western blot analyses. FB and Ba proteins, ranging from 7.5 to 240 ng/lane, were analyzed on Western blots for total protein and by specific antibody detection. Lanes 1 and 15 are protein ladders and lane 2 contains 60 ng of FB and of Ba. (**g** and **h**) FB (lanes 2-8) and Ba (lanes 9-14) were detected in (**g**) by total protein stain (entire lane intensity) at 700 nm (red); and in (**h**) with goat anti-human FB plus donkey anti-goat IRDye-800 (specific bands) at 800 nm (green). (**i** and **j**) Signal intensity plots of total lane protein for: (**i**) FB (r^2 = 0.9794); and (**j**) Ba (r^2 = 0.9949) versus lane amounts of FB and Ba proteins. (**k** and **l**) Signal intensity plots of band intensities detected by FB antibody for (**k**) FB (r^2 = 0.9928, ≤ 30 ng); and (**l**) Ba (r^2 = 0.9931, ≤ 60 ng) versus lane amounts of FB and Ba proteins. Coefficients of determination (r^2) were calculated using simple linear regression.

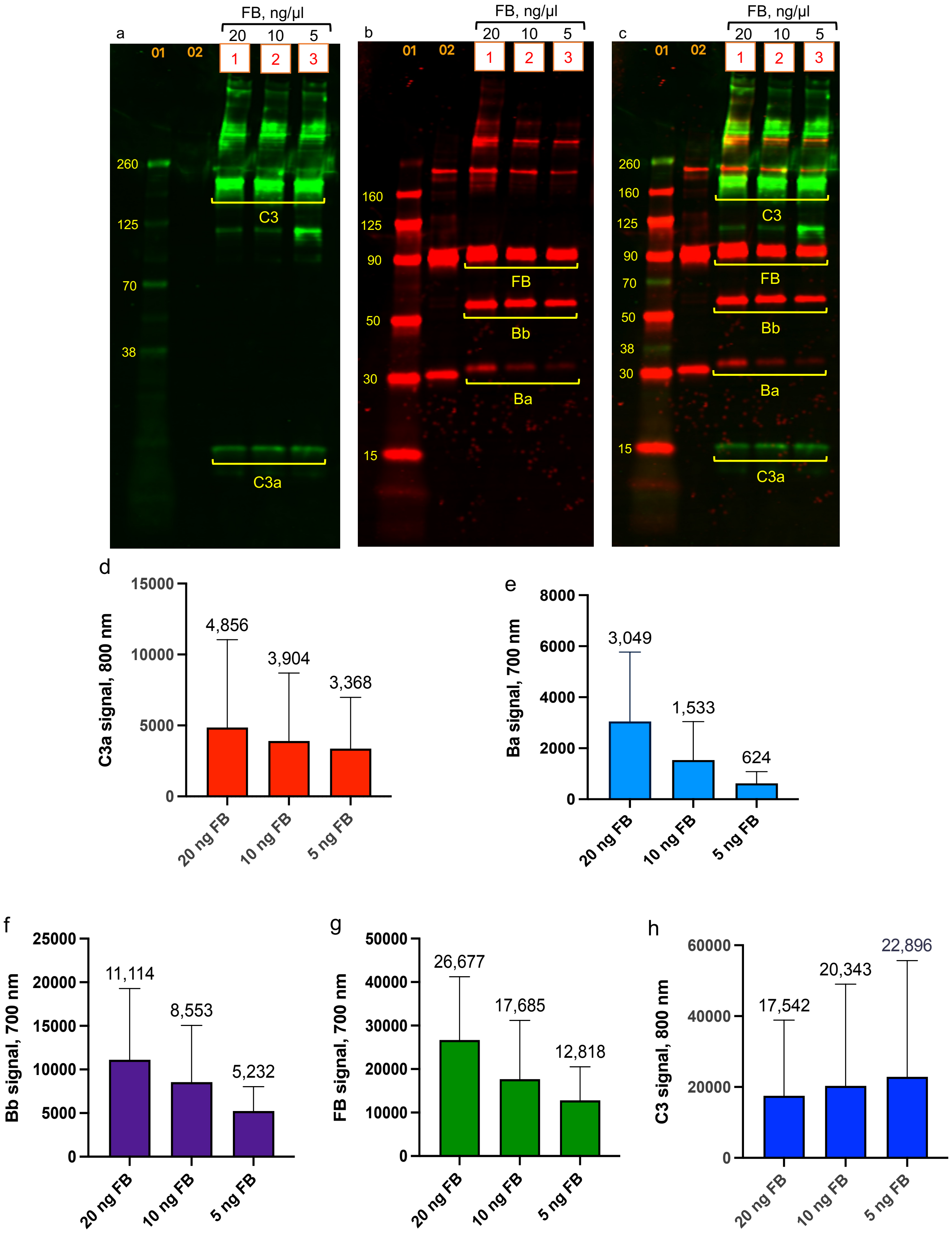

a
FB, ng/µl
20 10 5
01 02 1 2 3
260
125
70
38
C3
C3a
b
FB, ng/µl
20 10 5
01 02 1 2 3
160
125
90
50
30
15
FB
Bb
Ba
c
FB, ng/µl
20 10 5
01 02 1 2 3
260
160
125
90
70
50
38
30
15
C3
FB
Bb
Ba
C3a
d
C3a signal, 800 nm
4,856
3,904
3,368
20 ng FB
10 ng FB
5 ng FB
e
Ba signal, 700 nm
3,049
1,533
624
20 ng FB
10 ng FB
5 ng FB
f
Bb signal, 700 nm
11,114
8,553
5,232
20 ng FB
10 ng FB
5 ng FB
g
FB signal, 700 nm
26,677
17,685
12,818
20 ng FB
10 ng FB
5 ng FB
h
C3 signal, 800 nm
17,542
20,343
22,896
20 ng FB
10 ng FB
5 ng FB

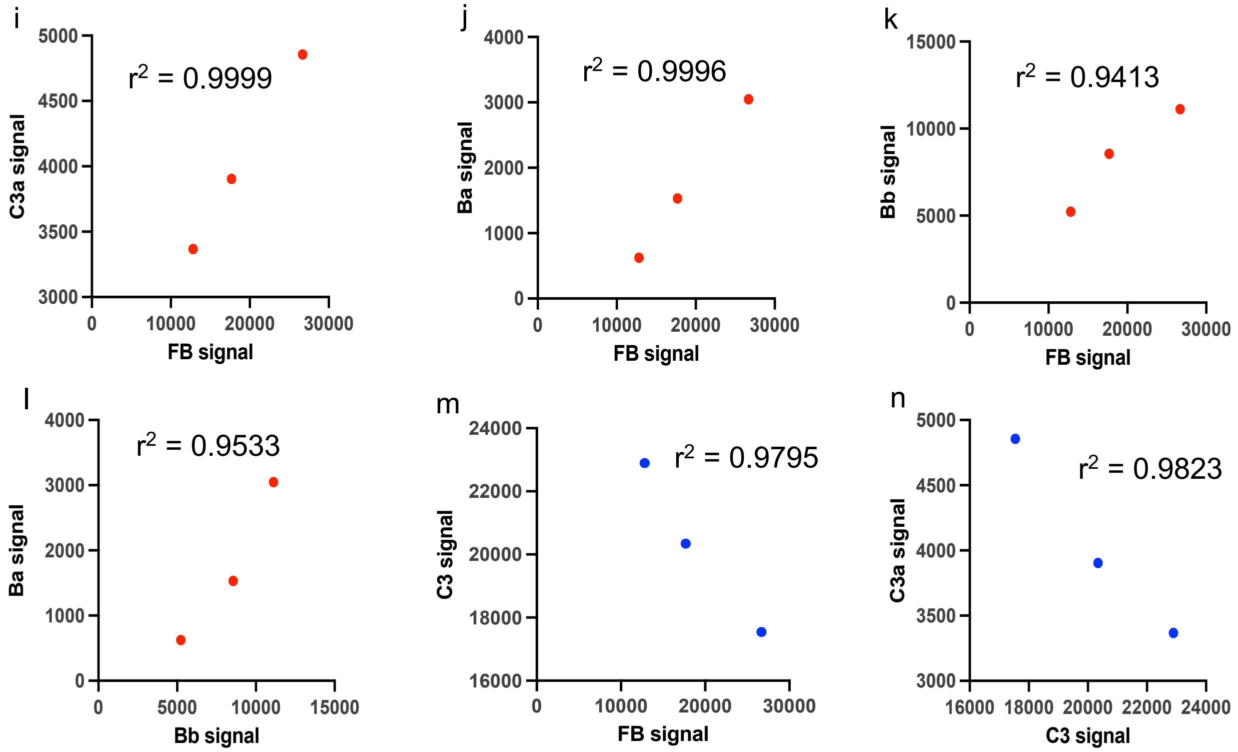

Supplementary Fig. S2. The extent of AP activation is FB-dependent. In the 3 reactions, concentrations of C3 (60 ng/µl) and FD (0.07 ng/µl) remained constant (at normal serum ratios), whereas FB concentrations were: (reaction 1) 20 ng/µl; (reaction 2) 10 ng/µl; and (reaction 3) 5 ng/µl. The blot was detected with: (**a**) rabbit anti-C3a + donkey anti-rabbit IRDye-800, green; (**b**) goat anti-FB + donkey anti-goat IRDye-680, red; and (**c**) both antibody pairs. The protein ladder is in lane 1; standards of FB (20 ng) and Ba (30 ng) are in lane 2; and the next 3 lanes contain the reaction samples, labeled with boxed numbers 1-3. Graphs show mean band signal intensities plus standard deviations (SD) from 3 experiments for: (**d**) C3a; (**e**) Ba; (**f**) Bb; (**g**) FB; and (**h**) C3. Mean band signal intensities were not statistically different by 2-way ANOVA with Tukey's multiple comparisons. Correlation plots show: (**i**) FB vs C3a, $p = 0.0065$; (**j**) FB vs Ba, $p = 0.017$; (**k**) FB vs Bb; (**l**) Bb vs Ba; (**m**) FB vs C3 (negative correlation); and (**n**) C3 vs C3a (negative correlation). Ba and C3a mean intensities were significantly correlated with the decreases in FB band intensities using Pearson's coefficient of determination (r^2).

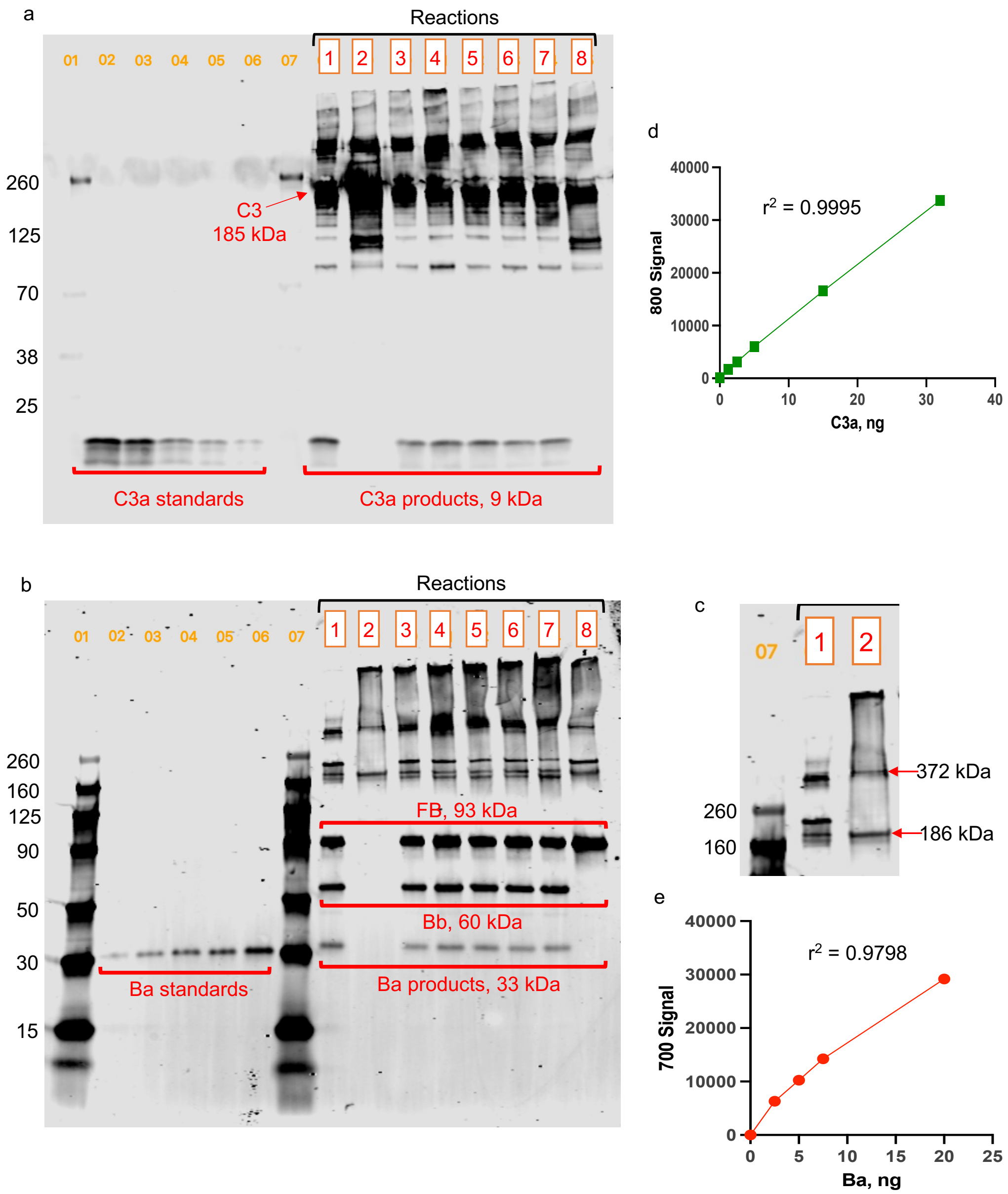

Supplementary Fig. S3. Measurements of C3a and Ba, in reactions with added HFB, by quantitative blot analysis. Standard curves using the known C3a and Ba protein quantities and their measured intensities were generated, and values from band intensities of unknown samples from reactions were interpolated. C3a standards ranging from 1.25 to 32 ng and Ba standards ranging from 2.5 to 20 ng are in lanes 2–6. Protein ladders are in lanes 1 and 7, and reaction samples are in lanes 8-15, labeled with boxed numbers 1-8. Western blot detection: (**a**) with rabbit anti-C3a + donkey anti-rabbit IRDye-800 (800 nm, grayscale); and (**b**) with goat anti-FB + donkey anti-goat IRDye-680 (700 nm, grayscale). (**c**) A section of the Western blot in (**b**) expanded to show HFB band sizes, possibly corresponding to multiple chains of FB. Standard plots of band intensity versus the range of known values for C3a (**d**) and Ba (**e**).

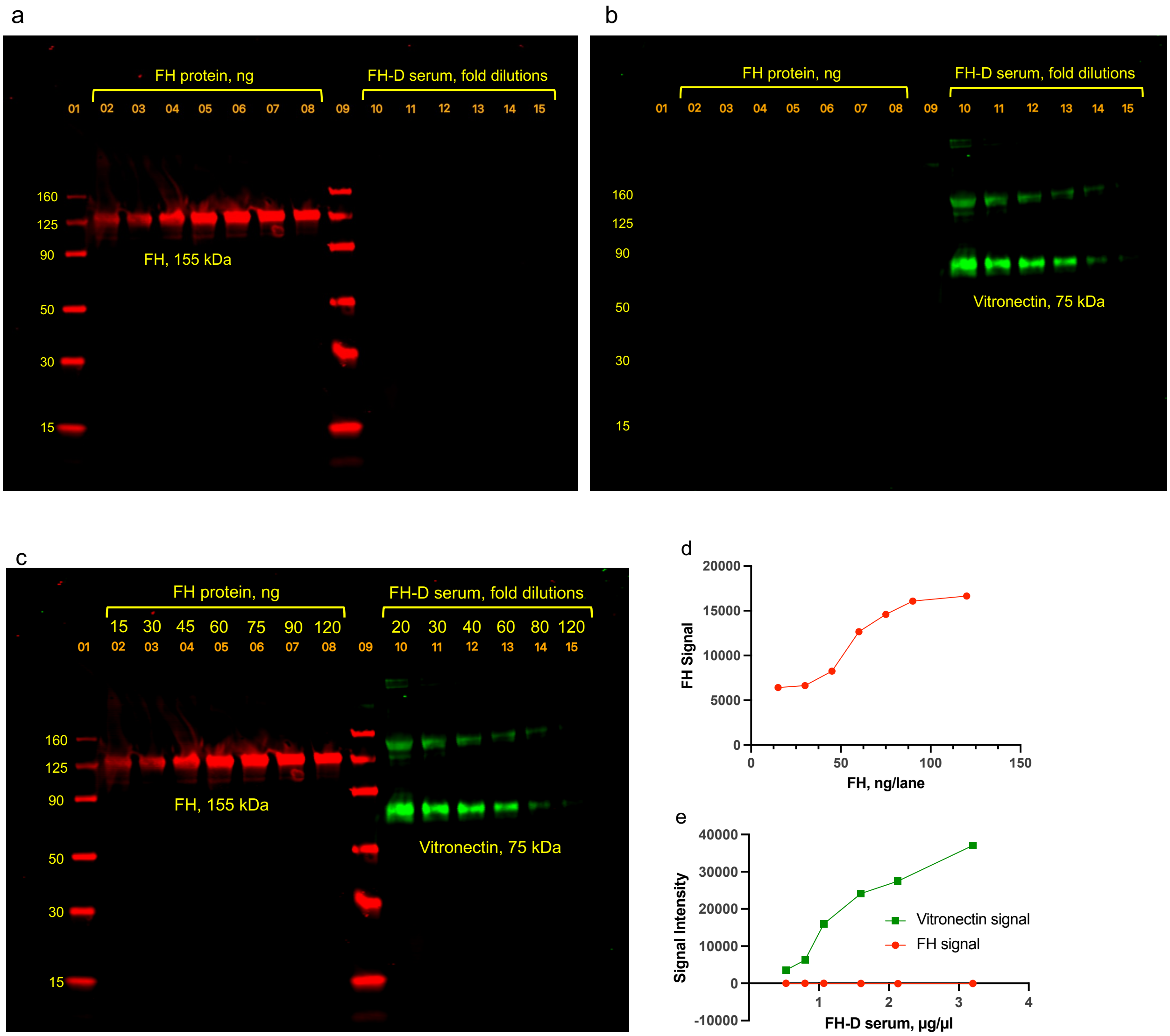

Supplementary Fig. S4. Detection of FH in FH-D serum. Gel lanes with 15, 30, 45, 60, 75, 90, and 120 ng of FH protein (lanes 2-8) and FH-D serum diluted 20-, 30-, 40-, 60-, 80- and 120-fold (protein concentrations: 3.2, 2.1, 1.6, 1, 0.8, and 0.5 µg/µl, lanes 10-15) were detected by Western blot techniques. The amount of FH-D serum, that was diluted 40-fold (lane 12), was equivalent to the amount of FH-D serum in reactions that were analyzed for AP activation products. The blot was imaged to detect: (**a**) FH using goat anti-FH plus donkey anti-goat IRDye-680 (red); (**b**) vitronectin using rabbit anti-vitronectin plus donkey anti-rabbit IRDye-800 (green); and (**c**) both FH and vitronectin. Vitronectin is plentiful in serum (240-540 mg/ml), and detection of vitronectin provided a method to visualize the FH-D serum-containing lanes that were blank for FH detection. Vitronectin (75 kDa) exists partially as disulfide-linked dimers, evident by the higher molecular weight bands of ~150 kDa. Lanes 1 and 9 contain the protein ladders. Plots of (**d**) FH detection signal versus FH protein amounts in lanes 2-8; and (**e**) FH detection signal (red) and vitronectin detection signal (green) versus FH-D serum concentration.

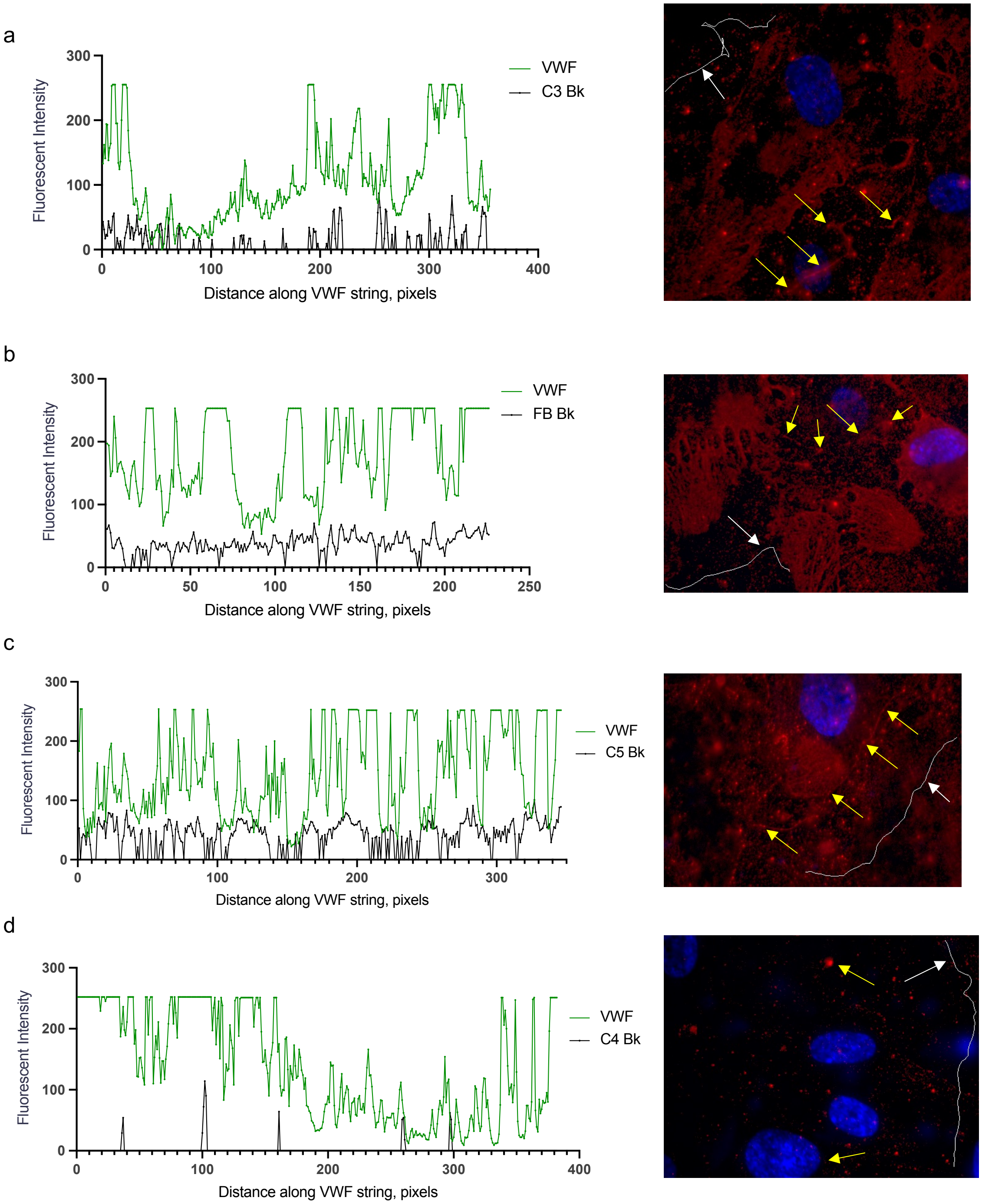

Supplementary Fig. S5. Background fluorescent intensity graphs and images. String background intensities in 647-detected (red) images were measured from identical string shapes, moved from original positions, and are shown in the 647-DAPI (blue) images as solid white lines indicated by a white arrow. The original position is marked by yellow arrows. In graphs, fluorescent intensities (y-axis) for VWF (green), measured from merged images (Fig. 4), and for 647-background strings (black), were plotted versus the ULVWF string lengths (x-axis). Shown are the background intensity plots and the images for: (**a**) C3; (**b**) FB; (**c**) C5; and (**d**) C4. Background = Bk.

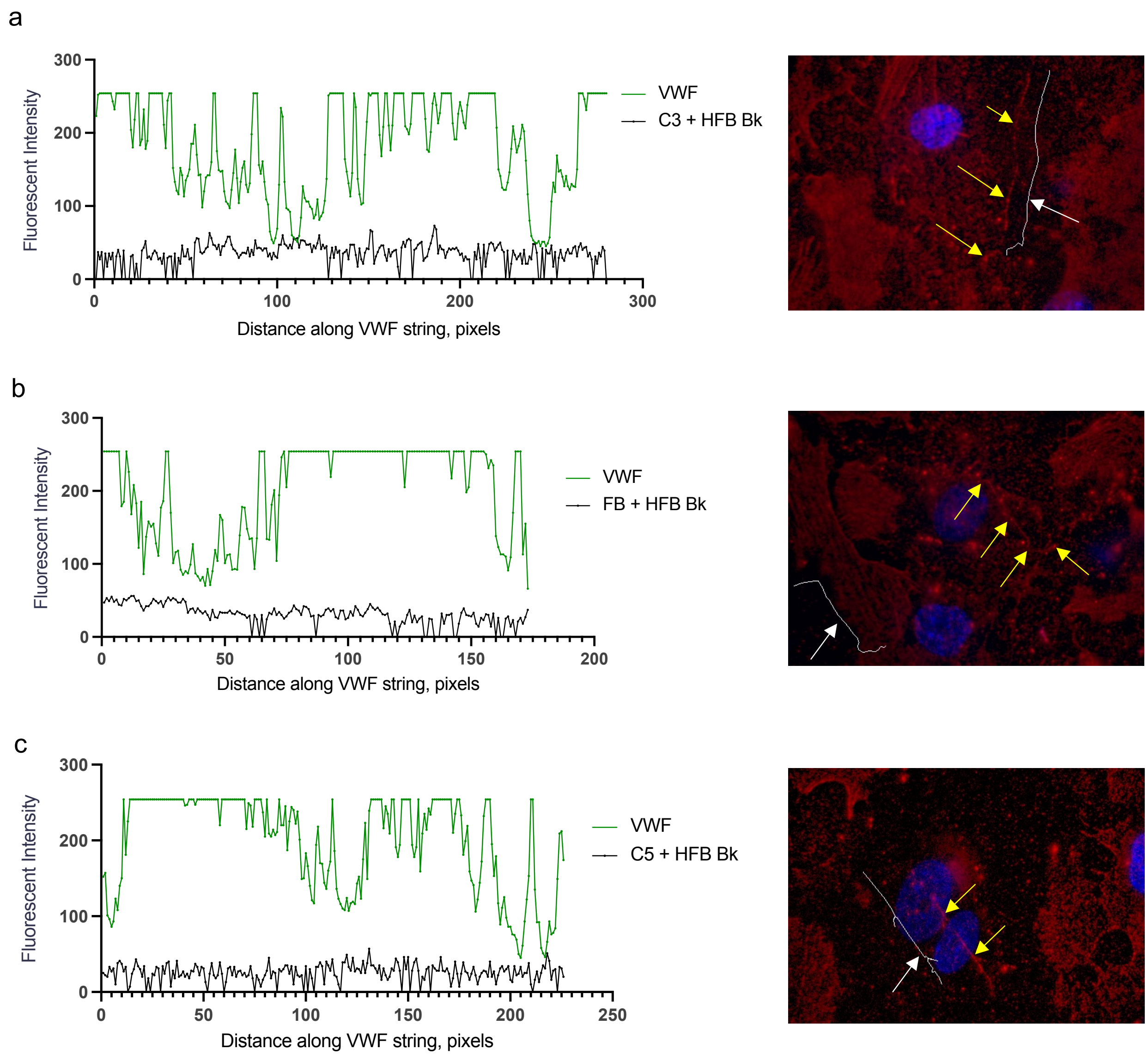

Supplementary Fig. S6. Background fluorescent intensity graphs and images. String background intensities in 647-detected (red) images were measured from identical string shapes, moved from original positions, and are shown in the 647-DAPI (blue) merged images as solid white lines indicated by a white arrow. The original position is marked by yellow arrows. In graphs, fluorescent intensities (y-axis) for VWF (green), measured from merged images (Fig. 5), and for 647-background strings (black), were plotted versus the ULVWF string lengths (x-axis). Shown are the background intensity plots and the images for: (**a**) C3, with presence of HFB; (**b**) FB, with presence of HFB; and (**c**) C5 with presence of HFB. Background = Bk.

Supplementary Table S1. Protein concentrations in reactions verifying FB-dependent AP activation

	Reaction concentrations, ng/µl			Normal serum	
	Reaction 1	Reaction 2	Reaction 3	Serum levels, µg/ml	C3 ratio
C3	60	60	60	1200	1
FB	20	10	5	200	6-fold lower
FD	0.07	0.07	0.07	1.4	857-fold lower

Concentrations of C3, FB, and FD in EGTA/$MgCl_2$ buffer with FB levels equal to, 2-fold lower, and 2-fold higher than FB in normal serum. Reaction 2 concentrations (shaded) are equal to ratios of C3, FB and FD in normal serum. Proteins in reactions were analyzed after 15 min at 37°C. The results are shown in Supplementary Fig. S2.

Supplementary Table S2. Data from the merged images of GMVEC-released complement proteins and secreted ULVWF strings

		Intensities of complement proteins detected attached to ULVWF strings		
Protein detected	ULVWF string length, µm	Mean intensity	Background intensity	Intensity per µm
C3	22.78	266.34	29.96	3538.03
FB	29.48	144.02	26.86	902.12
C5	47.30	219.89	34.67	1358.74
C4	48.39	40.64	10.30	240.21
C3 (+ HFB)	36.49	59.92	12.00	369.01
FB (+ HFB)	23.36	168.34	21.66	1092.53
C5 (+ HFB)	24.92	30.22	10.82	159.56

Data from the merged images of GMVEC-released complement proteins and secreted ULVWF strings shown in Figs. 4 and 5 and Supplementary Figs. S5 and S6. The intensity per µm of ULVWF string was calculated as the sum intensity of the complement protein along the ULVWF string, divided by the length of string, followed by subtraction of the background string value, calculated in the same manner. Background string intensities were measured from identical line shapes moved to another location within the same image (Supplementary Figs. S5, S6).
